# No impact of intravenous thrombolysis on post thrombectomy transcranial Doppler pulsatility index

**DOI:** 10.3389/fneur.2025.1681572

**Published:** 2025-11-21

**Authors:** Ammar Jumah, Savio Batista, Theja Yelam, Aaron Anderson, Erika Sigman, Jaydevsinh N. Dolia, Jonathan A Grossberg, Aqueel Pabaney, Pedro Martins, Raul G Nogueira, Diogo C. Haussen

**Affiliations:** 1 Department of Neurology, Emory University, Atlanta, GA, United States; 2 Department of Neurosurgery, Emory University, Atlanta, GA, United States; 3 Department of Neurology, University of Pittsburgh Medical Center, Pittsburgh, PA, United States

**Keywords:** acute ischemic stroke, transcranial Doppler, no-reflow, pulsatility index, cerebral autoregulation

## Abstract

**Background:**

While transcranial Doppler (TCD) studies are beneficial in evaluating macrovascular recanalization post mechanical thrombectomy (MT), they may have a role in estimating microvascular reperfusion. We aim to (1) evaluate if TCD-derived pulsatility index (PI) ratio is different in large vessel occlusion stroke (LVOS) patients that received intravenous thrombolysis (IVT), indicating potential beneficial impact on microvascular reperfusion, and (2) assess whether TCD metrics correlate with clinical and safety outcomes.

**Methods:**

This is a retrospective analysis of consecutive patients treated with MT for middle cerebral artery (MCA) M1 or intracranial internal carotid artery terminus (ICA) LVOS from January 2018 to June 2024. Patients with Expanded Thrombolysis in Cerebral Infarction (eTICI) grade 2c-3 reperfusion and high-quality TCD studies within 24 h of their procedure were included. Ipsilateral and contralateral mean flow velocity and PI were collected for the MCA and anterior cerebral artery (ACA). The primary outcome was the comparison of MCA PI ratios between IVT and non-IVT arms. Secondary outcome was the association between TCD-derived metrics and parenchymal hemorrhages (PH) or modified Rankin Scale at 90-days.

**Results:**

Of 1,962 patients, only 234 met the inclusion criteria. The median age was 65 (IQR 56–76) years, 45% were females, and 75.6% had MCA-M1 occlusions. Median ASPECTS was 8 (7–9), NIHSS 18 (13–22) and IVT was administered in 66 (28.2%) patients. Of all patients, 20 (8.5%) developed PH, 89 (50.3%) were independent, and 37 (20.9%) were dead by day 90. Adjusted analyses revealed no significant difference in MCA PI ratios between IVT and non-IVT groups (0.96 vs. 1.01, *p* = 0.36), and no significant associations between TCD metrics and PH or mRS at 90-days.

**Conclusion:**

In this retrospective study of LVOS with excellent reperfusion, no differences were found in PI ratio between IVT and non-IVT groups, and no associations between TCD parameters and PH or mRS at 90-days.

## 1 Introduction

Mechanical thrombectomy (MT) is the standard of care in select patients with acute ischemic stroke (AIS) due to large vessel occlusion (LVO) (1), leading to final successful reperfusion in >90% of patients (2). However, despite the advancement of devices and techniques, only one-third to one-half of all patients achieve functional independence at 90 days (1, 3). One potential explanation is microvascular reperfusion failure, often referred to as the “no-reflow” phenomenon, alongside other factors such as post-treatment re-occlusion, hemorrhagic transformation, and non-neurological complications during hospitalization (4–6).

Transcranial Doppler (TCD) ultrasonography is a ubiquitous and non-invasive imaging modality that allows for real-time analysis of cerebral hemodynamics (7). The pulsatility index (PI) is a commonly used hemodynamic parameter and is believed to reflect not only downstream resistance (8–10), but also autoregulatory mechanisms, cerebral perfusion pressure and arterial compliance (11). While TCD studies are commonly utilized to assess large artery recanalization post MT, its potential role in assessing the distal microvascular reperfusion is under investigation (5).

Our study aims to compare TCD-derived PI ratios in patients who underwent MT with and without intravenous thrombolysis (IVT), evaluating for a potential beneficial impact on microvascular reperfusion. Additionally, we explore the possible association of TCD-derived metrics, including mean flow velocity (MFV) and PI with clinical and safety outcomes.

## 2 Methods

### 2.1 Study setting and population

This is a retrospective analysis of a prospective maintained database including consecutive patients with AIS treated with MT at a single large comprehensive stroke center from January 2018 to June 2024. The following inclusion criteria were applied: (1) AIS secondary to a middle cerebral artery (MCA) M1 segment or intracranial internal carotid artery terminus (ICA-T), (2) MT within 24 h of last-known normal with an excellent reperfusion, defined as Expanded Thrombolysis in Cerebral Infarction (eTICI) grade 2c-3, and (3) TCD studies within 24 h of MT reperfusion. Patients were excluded if they had no TCD studies or if poor and/or absent acoustic temporal windows were observed. IVT included both alteplase and tenecteplase. This study was approved by the institutional review board and the need for informed consent was waived due to the retrospective nature of the study.

### 2.2 Transcranial Doppler

At our institution, the TCD studies are performed by experienced sonographers using a handheld 2-MHz probe as part of the Dolphin TCD system (Viasonix, Buffalo, NY) within a laboratory accredited by the Intersocietal Accreditation Commission. The TCD findings and reports were retrospectively reviewed and collected by a vascular neurology fellow with RPNI (Registered physician in neurovascular interpretation) certification (AJ). Uncertainties were adjudicated by a RPNI certified vascular neurologist (AA) and/or neurointensivist (ES). The MFV and PI of the MCA was recorded at a depth of 55–65 mm (corresponding to the M1 segment), and the ACA at a depth of 60–75 mm (corresponding to the A1 segment). To ensure accuracy, PI values were manually extracted from the waveforms, given that automated software measurements may occasionally yield erroneous values. Recordings for MFVs and PIs were documented for ipsilateral and contralateral sides to the stroke. MFV and PI ratios were calculated by dividing the ipsilateral metrics over the contralateral ones (MFV or PI). We also calculated ipsilateral MCA/ACA MFV ratio by dividing the ipsilateral MCA by the ipsilateral ACA MFV. Similarly, the contralateral MCA/ACA MFV ratio is determined by dividing the contralateral MCA by the contralateral ACA MFV. In our study, TCD metrics were analyzed at 24–36 hours of MT, as these metrics are significantly influenced by intracranial pressure secondary to cerebral edema that usually maximizes 2–5 days after stroke onset (12). MFV was automatically calculated by the TCD machine using peak systolic velocity (PSV) and end diastolic velocity (EDV) using the following formula.


$$\mathrm{MFV}=\frac{\mathrm{PSV}+2\mathrm{EDV}}{3}$$

### 2.3 Outcomes

The primary outcome was the effect of IVT status on the MCA PI ratio. Secondary outcomes were the correlation between MCA and ACA parameters (ipsilateral MCA PI, MFV ratio, and MCA/ACA ratio) with PH as well as modified Rankin Scale (mRS) shift at 90-days. Subgroup analyses utilizing ≥1.3 and ≥2.0 for MCA MFV ratio were pursued (13, 14). Functional independence was defined as mRS 0–2. PH was defined as either parenchymal hematoma-1 (PH1) or 2 (PH2) utilizing the Safe Implementation of Thrombolysis in Stroke-Monitoring Study (SITS-MOST) classification (15). A sensitivity analysis for the primary and secondary outcomes was performed in a cohort expanded to patients who underwent TCD studies within 36 h of reperfusion.

### 2.4 Statistical analysis

A database was created using Excel to store and analyze the collected data. For categorical covariates, frequency and proportion values were provided. Linear logistic regression models were performed to assess differences in TCD variables between IVT and non-IVT and PH and non-PH patients, while ordinal logistic regression was to evaluate their association with other secondary outcomes. Both models were adjusted for covariates including age, gender, hypertension (HTN), diabetes mellitus (DM), heart failure (HF), intracranial atherosclerotic disease (ICAD), and extracranial carotid disease. Results were reported as regression coefficients and odds ratios (OR). Parameter estimates, 95% confidence intervals (CIs), and *p*-values were reported. All statistical analyses were performed in R, Version 4.3.2 (R Foundation for Statistical Computing, Vienna, Austria). *p*-values smaller than 0.05 were considered to be statistically significant.

## 3 Results

### 3.1 Baseline variables

**Figure 1:**
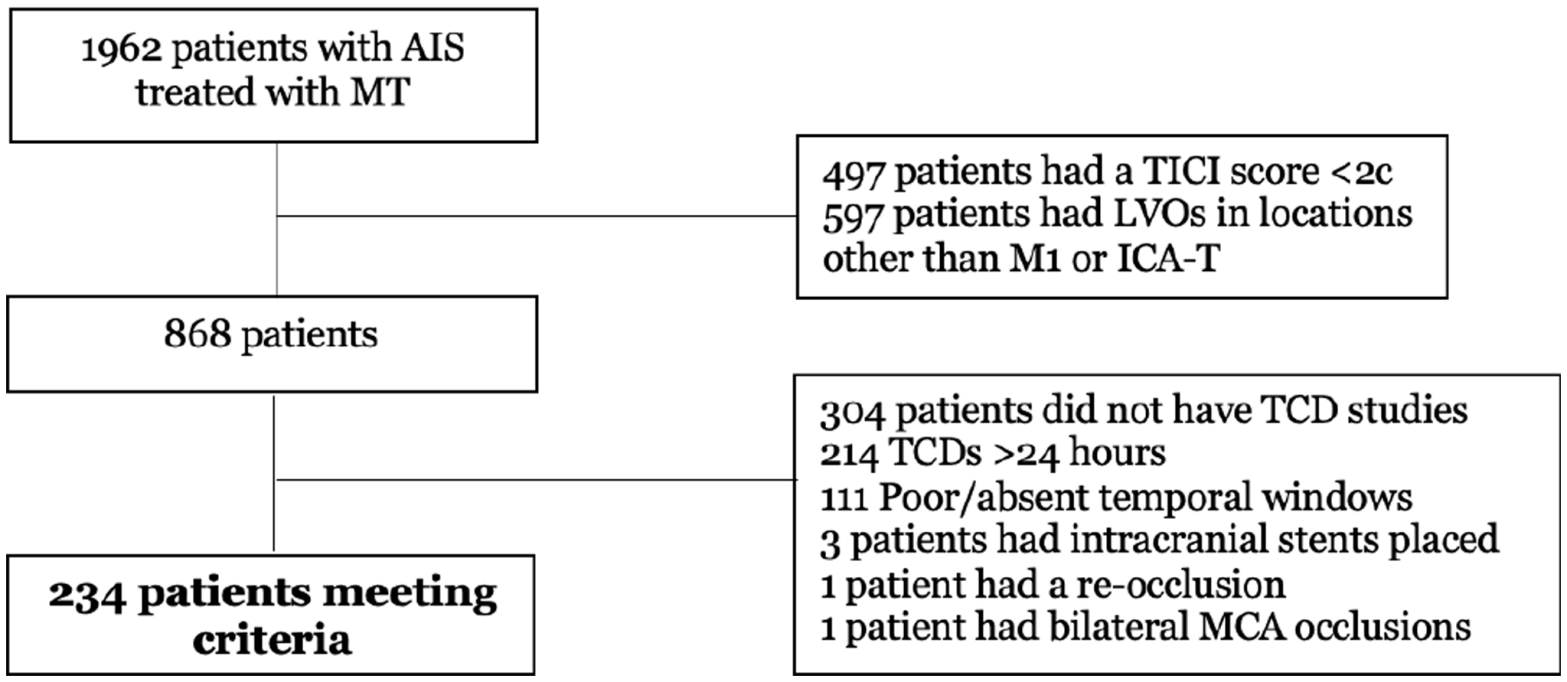
Study flowchart. AIS, acute ischemic stroke; LVO, large vessel occlusion; MT, mechanical thrombectomy; TCD, transcranial Doppler.

The study flowchart is shown in Figure 1. Of 1,962 patients treated within the study period, 868 had MCA-M1 or ICA-T occlusions and eTICI 2c-3, and 234 (11.9%) met the inclusion criteria. Median age was 65 (IQR 56–76) years, 105 patients (45%) were females, and 110 (47%) were black. Regarding the site of the occlusion, 177 patients (75.6%) had an MCA-M1 and 57 (24.4%) had an ICA-T occlusion. The Alberta Stroke Program Early CT Score (ASPECTS) and the National Institutes of Health Stroke Scale (NIHSS) were similar: 8 (IQR 7–9), and 18 (IQR 13–22), respectively. TCDs were performed at a median time of 13 h 17 min (IQR 6 h 55 min - 18 h 4 min) from recanalization. The rate of IVT was 28.2% (n = 66), and among those patients, 47 (71.2%) received alteplase and 19 (28.8%) received tenecteplase. Intra-arterial thrombolytics (alteplase) were administered in 2 (3%) patients in the IVT group and 1 (0.6%) in the non-IVT group. Of all patients, 20 (8.5%) developed PH. Functional independence and mortality at 90 days were 49.3 and 20.9%, respectively (Table 1). The median ipsilateral MCA PI was 1.19 (0.99, 1.43) and MCA MFV was 60.4 (45.5, 85.9).

**Table 1:** Baseline demographics.

Variable	All patients (*n* = 234)	IVT (*n* = 66)	Non-IVT (*n* = 168)	*p*-value
Age, median (IQR)	65 (56–76)	65.5 (59–76.75)	65 (55–76)	0.60
Male sex, *n* (%)	129 (55%)	35 (53%)	93 (55.4%)	0.71
Race, *n* (%)				
White	96 (41%)	27 (40.1%)	69 (41.1%)	0.95
Black	110 (47%)	31 (47%)	79 (47%)	0.96
Latin	3 (1.3%)	1 (1.5%)	2 (1.2%)	0.84
Asian	4 (1.7%)	1 (1.5%)	3 (1.8%)	0.88
Other	6 (2.6%)	2 (3%)	4 (2.4%)	0.78
Comorbidities, *n* (%)				
HTN	164 (70%)	50 (75.8%)	114 (67.9%)	**<0.05**
DM	59 (25.2%)	18 (27.3%)	41 (24.4%)	0.05
PAD	5 (2.1%)	1 (1.5%)	4 (2.4%)	**<0.05**
AFib	67 (28.6%)	15 (22.7%)	52 (31%)	**<0.05**
CHF	41 (17.5%)	9 (13.6%)	32 (19%)	0.08
HLD	67 (28.6%)	20 (30.3%)	47 (28%)	0.05
Smoking	42 (17.9%)	10 (15.2%)	32 (19%)	0.05
Pre-treatment SBP, median (IQR)	145 (130–167)	146 (91.5–137)	145 (128.25–169.75)	0.86
Pre-treatment DBP, median (IQR)	80 (73–95)	80.5 (75–90)	79.5 (71–95)	0.59
General anesthesia	68 (29.1%)	20 (30.3%)	48 (28.6%)	0.69
ASPECTS, median (IQR)	8 (7–9)	8.5 (7–10)	8 (7–9)	0.61
NIHSS, median (IQR)	18 (13–22)	18 (13–21.5)	18 (13–22)	0.72
Puncture-to-TCD, median (IQR)	14 h (7 h 45 min - 18 h 51 min)	13 h 11 min (9 h 8 min - 18 h 30 min)	14 h 23 min (6 h 46 min - 19 h 28 min)	0.88
Reperfusion-to-TCD, median (IQR)	13 h 17 min (6 h 55 min - 18 h 4 min)	12 h 16 min (8 h 8 min - 17 h 20 min)	13 h 36 min (5 h 42 min - 18 h 8 min)	0.90
IVT	66 (28.2%)		N/A	
Alteplase	47/66 (71.2%)		N/A	
Tenecteplase	19/66 (28.8%)		N/A	
IAT	3 (1.3%)	2 (3%)	1 (0.6%)	0.49
Stroke etiology, *n* (%)				
Cardioembolic	99 (42.3%)	27 (40.9%)	72 (42.9%)	0.75
Large vessel disease	28 (12%)	4 (6.1%)	24 (14.3%)	0.07
ICAD	23 (9.8%)	3 (4.5%)	20 (12%)	0.08
Dissection	4 (1.7%)	0	4 (2.4%)	0.20
Cryptogenic	50 (21.4%)	22 (33.3%)	28 (16.7%)	**<0.05**
Other	27 (11.5%)	9 (13.6%)	18 (10.7%)	**<0.05**
PH, *n* (%)	20 (8.5%)	10 (15.2%)	10 (6.0%)	0.14
mRS				
0	26/177 (14.7%)	9/49 (18.3%)	17/128 (13.4%)	0.47
1	44/177 (24.9%)	13/49 (26.5%)	31/128 (24.2%)	0.84
2	19/177 (10.7%)	5/49 (10.2%)	14/128 (10.9%)	1.0
Mortality	37/177 (20.9%)	8/49 (16.3%)	29/128 (22.7%)	0.41

AFib, atrial fibrillation; ASPECTS, Alberta Stroke Program Early CT Score; CHF, congestive heart failure; DBP, diastolic blood pressure; DM, diabetes mellitus; HLD, hyperlipidemia; HTN, hypertension; ICAD, intracranial atherosclerotic disease; IAT, intra-arterial thrombolysis; IVT, mRS; modified Rankin scale, intravenous thrombolysis; NIHSS, National Institute of Health Stroke Scale; PAD, peripheral arterial disease; PH, parenchymal hematoma; SBP; systolic blood pressure. Bold values indicate statistical significance.

### 3.2 Primary outcome

After adjustments, IVT administration was associated with a numerically lower MCA PI ratio (Median 0.96 [IQR, 0.86–1.08] vs. 1.01 [IQR, 0.87–1.13]), with an average 0.38-unit lower PI among IVT patients compared with non-IVT patients, though not reaching statistical significance (*p* = 0.36; Table 2).

**Table 2:** Linear logistic regression analysis: association between PI ratio with IVT and non-IVT groups.

TCD parameter	IVT, median (IQR)	Non-IVT, median (IQR)	Coefficient*	*p*-value*
MCA PI ratio	0.96 (0.86–1.08)	1.01 (0.87–1.13)	−0.38	0.36

IVT, intravenous thrombolysis; MCA, middle cerebral artery; PI, pulsatility index; TCD, transcranial Doppler. (MCA PI ratio was calculated by dividing ipsilateral PI by contralateral PI). *Adjusted for age, gender, HTN, DM, HF, ICAD, and extracranial carotid disease.

### 3.3 Secondary outcomes

There was no significant correlation between ipsilateral MCA PI, MCA MFV ratio, or MCA/ACA MFV ratio with functional outcome at 90 days (Table 3). In the linear logistic regression model, there was no significant correlation found between TCD metrics and the occurrence of PH (Table 4).

**Table 3:** Ordinal logistic regression analysis for mRS shift using TCD parameters.

TCD parameter	Coefficient*	*p*-value*
MCA MFV ratio	0.50	0.08
≥1.3	−0.13	0.68
≥2.0	−0.55	0.41
MCA ipsilateral PI	−0.044	0.35
Ipsilateral MCA/ACA MFV ratio	−0.044	0.20

ACA, anterior cerebral artery; MCA, middle cerebral artery; mRS, modified Rankin scale; PI, pulsatility index; TCD, transcranial Doppler. The MCA MFV ratio was calculated by dividing the ipsilateral MFV by the contralateral MFV. The ipsilateral MCA/ACA MFV ratio is determined by dividing the ipsilateral MCA by the ipsilateral ACA MFV. *Adjusted for age, gender, HTN, DM, HF, ICAD, and extracranial carotid disease.

**Table 4:** Linear logistic regression analysis for PH using TCD parameters.

TCD parameter	Coefficient*	*p*-value*
MCA MFV ratio	−0.41	0.32
≥1.3	−0.01	0.72
≥2.0	−0.01	0.34
MCA ipsilateral PI	−0.87	0.23
Ipsilateral MCA/ACA MFV ratio	−0.43	0.35

ACA, anterior cerebral artery; MCA, middle cerebral artery; PH, parenchymal hematoma; PI, pulsatility index; TCD, transcranial Doppler. *Adjusted for age, gender, HTN, DM, HF, ICAD, and extracranial carotid disease.

### 3.4 Sensitivity analysis

In a sensitivity analysis including patients with TCD studies performed within 36 h (*n* = 267), there was no statistical difference between MCA PI ratio among patients who received IVT versus those who did not (data not shown). There was also no correlation between functional outcomes at 90 days with any of the TCD metrics (data not shown).

## 4 Discussion

In this retrospective study of a homogenous LVOS patient population (ICA-T or M1 occlusions with excellent reperfusion), no difference in MCA PI ratio between IVT versus non-IVT patient groups was observed. Additionally, there was no clinical correlation between TCD parameters (i.e., ipsilateral MCA PI, MCA MFV ratio, and ipsilateral MCA/ACA ratio) and the development of PH or functional outcomes at 90-days.

At the microvascular level, the no-reflow phenomena in AIS arise from a cascade of pathological events, including downstream micro-plugging by fibrin-rich aggregates, leukocytes and platelets, alongside luminal narrowing caused by pericyte contraction, astrocytic end-feet edema, dysfunction of endothelial cells (16, 17), and neutrophil-related capillary stalling (18). This phenomenon has been proposed as one plausible explanation for the lack of functional independence in patients who otherwise had a successful recanalization (4, 19). This phenomena has also been observed in the venous circulation. In one study, inducing an MCA occlusion triggered a cascade of leukocyte margination and adhesion in postcapillary venules, leading fibrin deposition, ongoing thrombi formation, and leukocyte extravasation—despite recanaliation of the occluded vessel (20). A meta-analysis of eight studies showed significantly lower odds of functional independence at 90-days when the no-reflow was observed (OR, 0.21; 95% CI, 0.15–0.31) (4). However, it is worth mentioning that the definition and quantification methods of the no-reflow varied across the literature. For instance, some studies considered a delay in time to maximum (Tmax) of >2 s on magnetic resonance perfusion (MRP) imaging to reflect a lack of reperfusion (21, 22), while others have considered Tmax >6 s utilizing computed tomography perfusion (CTP) as a more accurate threshold (23). Moreover, some studies have adopted interval change in lesion volume, reflected by the difference between Tmax estimated by CTP before and after intervention (i.e., tissue optimal reperfusion) (24). In the same systematic review, only two studies utilized TCDs to assess the microvascular status (5, 25), in which one study included TCD studies performed within 72 h of MT (5), a timeframe that may be unreliable in assessing the microvascular reperfusion due to the effects of evolving edema and raising intracranial pressure. The no-reflow is usually assessed using imaging modalities such as CTP (23), MRP (26), and single-photon emission computed tomography (SPECT) (27). Neither prior studies nor ours have validated TCD findings against these imaging modalities to confirm the failure of microvascular reperfusion.

Approximately one-third of patients who had a successful recanalization have been shown to have continued hypoperfusion on follow up imaging (4), suggesting that a successful recanalization does not always equate successful reperfusion (16). Accruing evidence, as demonstrated by a recent meta-analysis, indicates that intra-arterial thrombolysis (IAT) in patients with near-complete or complete recanalization may lead to improved clinical outcomes (6). It is hypothesized that adjunctive IAT post MT lyse thrombi in the microvasculature for an added benefit after the intervention (28). We hypothesized that PI would be a surrogate and would be lower due to the positive effects on distal microvascular resistance; however, in the present study, we did not observe differences in the MCA PI ratio between IVT and non-IVT patients.

We did not observe any associations between TCD parameters and the occurrence of PH. We could not corroborate previous studies that showed a direct correlation between MFV and hemorrhagic conversion. A recent meta-analysis showed that a MFV index (i.e., MFV of ipsilateral MCA divided by contralateral MCA) ≥ 1.3 was associated with a higher risk of hemorrhagic conversion after AIS (RR, 1.97; 95% CI, 1.28–3.03, *p* = 0.002) (13). Interpretation of the results should be approached with caution as velocity values were extrapolated from studies that included TCDs performed up to 48 h post MT and were limited to patients with reperfusion of ≥2b and M1-MCA LVOS only. Previous observational studies using SPECT have shown that a 2-fold increase in MCA blood flow velocity post carotid revascularization procedures predicts the development of postoperative hyperperfusion syndrome (14, 29). In our study, the ratio of ipsilateral to contralateral MCA MFV of ≥2 was not found to be associated with post-procedural PH.

The neutral results of our study could be attributed to the relative rarity of the no-reflow phenomena. It is also important to note that although PI is known to directly reflect distal microvascular resistance, multiple studies have challenged this concept. In one study, the authors show that PI not only increased with hypocapnia challenge, which induces vasoconstriction and increases distal microvascular resistance, but also in intracranial hypertension following the onset of an induced plateau wave where vasodilation occurs, raising intracranial pressure with a corresponding reduction of distal microvascular resistance (11). The authors suggest that it rather represents a relationship between cerebral perfusion pressure, pulse amplitude of arterial pressure, heart rate, compliance of the arteries, and cerebrovascular resistance. Therefore, the results based on TCD alone should be interpreted with caution, as its role in microvascular reperfusion remains to be uncertain.

While our study offers valuable insights, several limitations need to be acknowledged. The retrospective nature of this study may have introduced bias. Moreover, to capture early microvascular changes, TCDs should be ideally performed promptly after recanalization and before cerebral edema develops, possibly altering its values. Also, obtaining a TCD study at a single time point might not fully capture the microvascular injury cascade. Instead, conducting follow-up studies after the index study would provide greater insight into PI changes over time. Importantly, PIs are usually affected by variables that are challenging to be controlled, such as carbon dioxide, respiratory rate, antihypertensives, systolic and diastolic blood pressure, heart rate and cardiac output. Another limitation is that our dataset does not include information regarding the severity of cerebral microangiopathy. Differences in distal small vessel disease burden between groups might have influenced PI. Additionally, it lacks data on post-procedural blood pressure levels and pharmacotherapy, both of which could have impacted the results. Whether TCD-related parameters reflect the microcirculatory reperfusion remains to be determined.

## 5 Conclusion

In this retrospective study of LVOS with excellent reperfusion, no differences were found in PI ratio between IVT and non-IVT groups, and no associations between TCD parameters and 90-day modified Rankin scale and PH were observed.

## Data Availability

The original contributions presented in the study are included in the article/Supplementary material, further inquiries can be directed to the corresponding author/s.
