# Supplementary material for: No impact of intravenous thrombolysis on post thrombectomy transcranial Doppler pulsatility index
Source: Front Neurol. 2025 Nov 21;16:1681572. doi: 10.3389/fneur.2025.1681572 (PMC12679829; doi:10.3389/fneur.2025.1681572)
Supplement: Supplementary file 1 [file Table_1.docx]

**Supplementary Material of Post Thrombectomy Transcranial Doppler Parameters and Microvascular Reperfusion**

| 1. Table S1. Binary logistic regression analysis: Association between PI ratio with IVT and non-IVT groups | | | | |
| --- | --- | --- | --- | --- |
| TCD Parameter | **IVT,**  median (IQR) | **Non-IVT,**  median (IQR) | **Coefficient** | **P**-**Value** |
| MCA PI ratio | 0.95 (0.84 - 1.08) | 1.0 (0.87 - 1.13) | -0.41 | 0.53 |

| 1. Table S2. Ordinal logistic regression analysis for mRS shift using TCD parameters | | | |  |
| --- | --- | --- | --- | --- |
| TCD Parameter | **Coefficient** | | **P-value** |  |
| MCA Metrics | | | |  |
| MCA MFV Ratio | | -0.04 | 0.62 |  |
| MCA Ipsilateral MFV | | 0.0004 | 0.90 |  |
| MCA Contralateral MFV | | 0.01 | 0.30 |  |
| MCA PI Ratio | | -0.42 | 0.54 |  |
| MCA Ipsilateral PI | | -0.55 | 0.14 |  |
| MCA Contralateral PI | | -0.44 | 0.30 |  |
| ACA Metrics | | | |  |
| ACA MFV Ratio | | -0.07 | 0.69 |  |
| ACA Ipsilateral MFV | | 0.014 | 0.02 |  |
| ACA Contralateral MFV | | 0.007 | 0.26 |  |
| ACA PI Ratio | | -0.13 | 0.78 |  |
| ACA Ipsilateral PI | | | -0.45 | 0.19 |
| ACA Contralateral PI | | | -0.54 | 0.12 |
